# Harvesting energy from low-frequency excitations through alternate contacts between water and two dielectric materials

**DOI:** 10.1038/s41598-017-17522-8

**Published:** 2017-12-07

**Authors:** Jian Yu, Enze Ma, Tianwei Ma

**Affiliations:** 1 Department of Civil & Environmental Engineering, University of Hawaii at Mānoa, Honolulu, HI 96822 USA; 2 Department of Neuroscience, Duke University, Durham, NC 27708 USA; 3 College of Engineering, University of Hawaii at Mānoa, Honolulu, HI 96822 USA

## Abstract

Recent studies have demonstrated the benefits of water-dielectric interfaces in electrostatic energy harvesting. Most efforts have been focused on extracting the kinetic energy from the motions of water drops on hydrophobic surfaces, and thus, the resulting schemes inherently prefer cases where the water drops move at a high speed, or vibrate at a high frequency. Here we report a method for directly harvesting ambient mechanical energy as electric potential energy through water droplets by making alternate contacts with CYTOP and PTFE thin films. Because CYTOP and PTFE acquire significantly different surface charge densities during contact with water, such a difference can be utilized to effectively generate electricity. We demonstrate this concept using prototype devices fabricated on silicon substrates with a simple procedure. In the experiments conducted, a water drop of 400 *μ*L alone could generate a peak open-circuit voltage of 42 V under a 0.25 Hz vibration. Under a 2.5 Hz vibration, the peak open-circuit voltage reached 115 V under an external bias of 8 V. The demonstrated efficiency is orders of magnitude higher than those of existing devices of similar dimensions.

## Introduction

Advances in low-power electronics and distributed systems have stimulated significant interests in small-scale power supply systems that generate electricity from ambient environmental energy sources. There have been numerous studies on electricity generation from various sources, such as waste heat^1,2^, ambient mechanical energy^3–6^, salinity differences^7–9^, contact electrification^10,11^. Recently, the rich electrostatic phenomena at liquid-solid interfaces have spurred renewed interest in harvesting mechanical energy through electrostatics. It has been demonstrated that motions of water on a hydrophobic surface of a conductive or semiconductive material modify the electric double layer at the interface, which can be utilized for energy harvesting^12–15^. When the solid material is a dielectric, a similar phenomenon also occurs. Studies have demonstrated that repetitive contacts between a liquid droplet and an uncharged polymer film electrify the film, turning it into an electret^16–19^. If a conductive liquid droplet is placed on the polymer electret, the well-known electrostatic induction process will take place. The charge in the electret will attract the opposite charge in the droplet to the interface and repel the similar charge away. Consequently, the distribution of the surface charge in a nearby conductor will also change.

Methods have been proposed recently to utilize such electrostatically induced change in the distribution of charges to harvest external energy as electric current. More specifically, ambient mechanical energy is harvested through continuous control of the charge that is attracted to a liquid-solid interface, thereby creating an alternate current^18,20–22^. This control is achieved by manipulating the contact area at the interface through either deformations of a water drop^22,23^ or separation of a droplet from a polymer, and in the latter case, the contact area becomes null at complete separation^18,19^. Because the resulting electric current depends on the kinetic energy of the droplet, existing schemes are inherently suited for cases involving fast excitations. Note that fundamentally, electrostatic energy harvesting relies on the work done by external energy against electrostatic forces so that the work is converted to electric potential energy. It is, therefore, more efficient to directly harvest the electric potential energy at its peak^20,24^ than to harvest it through the kinetic energy. Furthermore, continuously changing the contact area requires either a precise, consistent control of the deformations of individual droplets or a source that continuously produces droplets for separation, but both are difficult to achieve under ambient vibrations.

Here we report an electrostatic harvesting approach which takes advantage of water moving across dielectric materials that possess significantly different surface charge densities. Such difference enables strong electrostatic induction and thus, leads to a high harvesting efficiency. The larger the difference in surface charge density, the more the harvested energy. Because CYTOP and PTFE respond to contact electrification very differently, a hydrophobic surface created with them will be electrified at different levels if brought into contact with water^16,17^. As a result, when a water drop moves across the CYTOP and PTFE surface, a fixed amount of charge is induced without the need for physical changes of the contact area. More importantly, the proposed method utilizes the strong electrostatic induction in the water drop due to the electrical double layer formed at the interface. Therefore, it is more effective than existing methods that are based on the much weaker electrostatic induction in the substrate. Also, unlike in existing methods, in which the charge of the drop is not delivered to external circuits, in our method, the water drop possesses a dual function as both an electrode and a passive switch, leading to the direct harvesting of the peak electric potential energy. This method not only results in simple device architecture but also allows for the use of schemes based on variable capacitors to improve its performance. Using prototype devices, we demonstrate the effectiveness of this approach in scavenging energy from low-level and low-frequency ambient vibrations. Each device was fabricated on a doped silicon substrate with one side coated by a thin, dielectric oxide layer. CYTOP and PTFE were used to create the two-region hydrophobic surface on which a 400 *μ*L water drop was free to move. Under a 0.25 Hz vibration, the device in which the CYTOP and PTFE regions were fabricated with similar thicknesses could generate a peak open-circuit voltage of 42 V. When the CYTOP and PTFE regions were fabricated with significantly different thicknesses, i.e. 8 *μ*m and 0.5 *μ*m for CYTOP and PTFE, respectively, the device could generate a peak open-circuit voltage of 115 V under an 8 V external bias when the device was driven under a 2.5 Hz vibration. It should be noted that the focus of this study was placed on the energy harvesting mechanism. Practical issues, such as optimization of device architecture, design of supporting circuitry, packaging, etc., were not considered.

## Results

### Working mechanism

**Figure 1:**
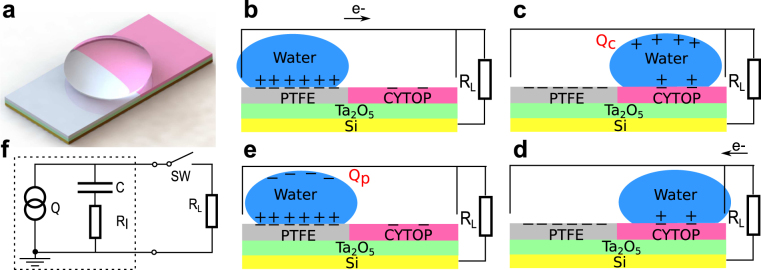
Working principle of the prototype harvester. (**a**) A 3D rendered model of the device. (**b**–**e**) Schematics of an energy harvesting cycle. (**b**) A water drop on PTFE with the electric load connected to the device. (**c**) Positive charge induced in the water drop when it was driven from PTFE to CYTOP. (**d**) Electrical power delivered to electric load connected to the device. (**e**) The drop driven to PTFE. (**f**) The equivalent circuit model of the generator. Q, C, and *R*_*I*_ represent the induced charge, the equivalent capacitor, and the internal resistive loss, respectively, and *Q* = *Q*_*p*_ = −*Q*_*c*_, where subscripts *p* and *c* represent values for cases of the water drop being on PTFE and CYTOP, respectively.

The working principle of the proposed method is explained using Fig. 1. A 3D rendered model of a prototype harvester fabricated in this study is shown in Fig. 1a. The process of electricity generation is illustrated in Fig. 1b–e. The harvester was fabricated on a doped silicon substrate, which functioned as a fixed electrode. One side of the substrate was coated with a layer of tantalum pentoxide (Ta_2_O_5_), 300 nm thick. Tantalum pentoxide was coated with PTFE and CYTOP, each covering half of the surface. A free-standing water drop on the hydrophobic surface functioned as a moving electrode. The oxide layer was necessary to prevent electric leaking due to pin-holes in the hydrophobic coatings. Due to contact electrification, the initial contacts between the water drop and the electrically neutral surface will electrify the surface so that both PTFE and CYTOP are negatively charged. If multiple contacts are made, the charge will quickly saturate at very different values. The charge in PTFE and CYTOP will be quasi-permanent, making the two sections behave as electrets. Subsequent motions of the water drop on the surface will not only help maintain the charge in the hydrophobic coatings but also invoke electrostatic induction. If an electrically neutral water drop is placed on the charged PTFE surface, the positive charge in the drop will be attracted to the interface to form an electric double layer^25^, and the negative charge is repelled away from the interface, creating a negative potential relative to the silicon substrate, which will create an electrical current through the metal contact as illustrated in Fig. 1b. While CYTOP and PTFE generate similar water contact angles, and thus similar contact areas, the saturated surface charge density of CYTOP is significantly lower. If the drop subsequently moves to the CYTOP surface, the electric double layer will change accordingly. Therefore, the potential of the drop will become positive relative to the silicon substrate (Fig. 1c). An opposite current can be generated if the drop is connected to the external load as shown in Fig. 1d. The subsequent motion of the drop back to the PTFE surface will reestablish the original electric double layer and create a negative potential of the drop (Fig. 1e). Therefore, an alternate current can be generated when the drop continuously moves across the surface. Note that electrostatic induction will also take place in the silicon substrate due to the quasi-permanent surface charge. Therefore, the generated current theoretically includes the induced charge in the substrate. However, because the thickness of the double layer formed at the water-polymer interface is on the order of 10 nanometers^26^, much smaller compared to the distance between the substrate and the surface charge, which is on the order of micrometers, the electrostatic induction in the water drop is the dominant mechanism in the process as confirmed experimentally. The potential energy of the induced charge on the water drop can be modeled as the energy stored in an equivalent capacitor, with the water drop and the substrate as the electrodes. Therefore, the effect of electrostatic induction is equivalent to that of a charge source, which provides a fixed amount of charge to the equivalent capacitor. A schematic circuit model of the generator is shown in Fig. 1f, where for simplicity, both the energy loss and the electrical load are assumed to be resistive with resistances of *R*_*I*_ and *R*_*L*_, respectively. The charge transferred to the capacitor is equal to the electrostatically induced charge on the water drop, which can be estimated as

1 $$Q=-{Q}_{p}={Q}_{c}={\sigma }_{c}{A}_{c}-{\sigma }_{p}{A}_{p}$$

where *Q*, *σ* and *A* represent the induced charge, the surface charge density, and the contact area, respectively. Subscripts *p* and *c* indicate the values for PTFE and CYTOP, respectively. Due to the similar contact angles of PTFE and CYTOP^27,28^, the static contact areas were approximately the same, i.e. *A*_*p*_ ≈ *A*_*c*_. Because of the much higher relative permitivity of tantalum pentoxide, i.e. 25 as compared to 2.2 for CYTOP and 1.93 for PTFE, and its much lower thickness, the contribution of the Ta_2_O_5_ layer to the total capacitance between the water drop and the substrate is negligible. Therefore, the capacitances can be calculated as *C*_*p*_ = *ε*_0_ *ε*_*p*_ *A*_*p*_/*d*_*p*_ and *C*_*c*_ = *ε*_0_ *ε*_*c*_ *A*_*c*_/*d*_*c*_, where *ε*_0_ is vacuum permittivity, *ε*_*p*_ and *ε*_*c*_ are the relative permittivities of PTFE and CYTOP, respectively, *d*_*p*_ and *d*_*c*_ represent the thicknesses of the hydrophobic coatings. The total harvested energy in one cycle can be written as

2 $$E=(\frac{{d}_{p}}{2{\varepsilon }_{0}{\varepsilon }_{p}{A}_{p}}+\frac{{d}_{c}}{2{\varepsilon }_{0}{\varepsilon }_{c}{A}_{c}})\,{Q}^{2}$$

### Device performance

#### Two hydrophobic regions with similar thicknesses

**Figure 2:**
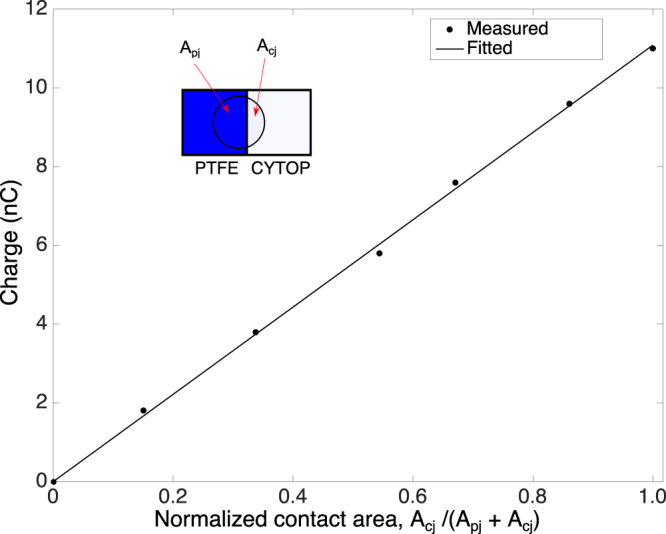
Relationship between the charge induced on a 400 *μ*L water drop and the normalized contact area. *A*_*pj*_ and *A*_*cj*_ represent the partial contact areas of the drop that belong to the PTFE and CYTOP sides, respectively.

Here we present the results from a harvester, in which the thicknesses of the PTFE and CYTOP were 6.6 *μ*m and 10 *μ*m, respectively. A tungsten contact was used at each side for the connection to the external circuit. Multiple contacts were first made between water and the CYTOP and the PTFE surface to create a charged surface. A fresh water drop was then deposited respectively onto the surfaces and the charge induced in the drop was measured with an electrometer using the silicon substrate as the reference. In this study, PTFE and CYTOP acquired −10.7 nC/cm^2^ and −1.8 nC/cm^2^, respectively. In order to study the induced charge when a water drop moves across the junction, a 400 *μ*L water drop was first placed on the PTFE surface with the induced charge zeroed by connecting a short circuit between the drop and the silicon substrate. The drop was then moved across the junction towards the CYTOP surface with the induced charge measured at different locations. The results are shown in Fig. 2. The linear relationship is consistent with the model indicated by Eq. (1). The difference in surface charge densities of the two surfaces can be determined from Fig. 2 to be 9.4 nC/cm^2^, which is consistent with that (8.9 nC/cm^2^) obtained from direct measurements of the two surface charge densities.

**Figure 3:**
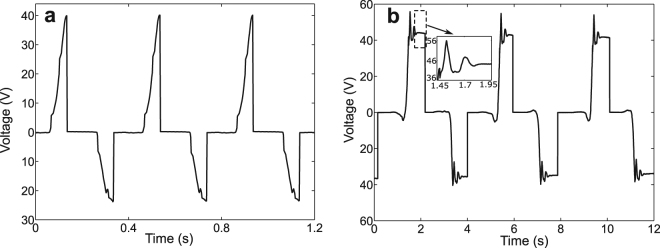
Voltages measured between the water drop and the substrate. (**a**) The device driven by a 2.5 Hz excitation and the electric load connected via tungsten contacts. (**b**) The device driven manually at 0.25 Hz and the electric load connected via manual switches.

**Figure 4:**
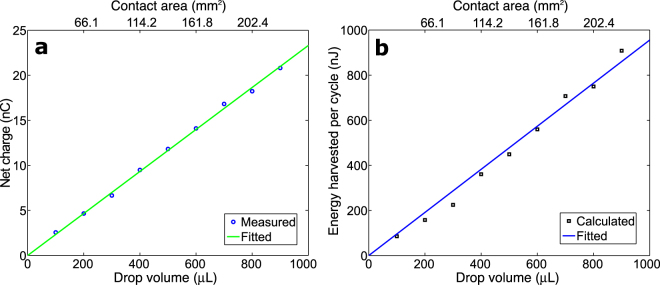
Effect of drop volume on device performance. (**a**) Net charge generated. (**b**) Energy harvested per cycle.

To evaluate its effectiveness in harvesting energy from low-frequency vibrations, the harvester was first driven by a 2.5 Hz excitation, a frequency close to those of vibrations induced by human walking. A 400 *μ*L water drop and a resistor of 1 MΩ were used in the experiments. The contact areas were calculated to be *A*_*p*_ ≈ *A*_*c*_ ≈ 1.14 cm^2^. The capacitances when the water drop was on CYTOP and PTFE were measured statically to be 0.23 nF and 0.28 nF, respectively. Figure 3a shows the voltages measured between the water drop and the substrate. Power was delivered to the resistor whenever the water drop moved across the surface, i.e. twice in a cycle. When the water drop was driven from the PTFE to the CYTOP surface, the voltage increased from 0 V to 40 V immediately before the resistor was connected. When the drop was driven back to the PTFE surface after releasing the positive potential to the resistor, the voltage changed from 0 V to −23.5 V. The asymmetry of the positive and negative peak values was due to the different capacitances associated with the two regions. Such difference in capacitance resulted from several factors associated with the two regions, including differences in thicknesses, dielectric constants, and dynamic contact areas, etc. In order to evaluate its performance under excitations of ultra-low driving frequencies, such as those of vibrations of large, flexible structures^29^, the harvester was manually driven at a very low frequency, about 0.25 Hz (Supplementary Fig. S1). Figure 3b shows the output voltages of the harvester. A manual switch was used to close the circuit so that the charge could be delivered to the load. When the water drop moved from the PTFE to the CYTOP surface, the open-circuit voltage was about 42 V while the corresponding charge was measured to be 9.5 nC, which is consistent to the estimated value of 10.1 nC based on Eq. (1). The open-circuit voltage stayed at the same value when the water drop was held in the same place. This confirms the capacitive behavior of the device. After the drop was discharged, the water drop was moved back to the PTFE surface. An open-circuit voltage of −34 V was then obtained. The corresponding charge was measured to be −9.5 nC. The difference in the capacitances associated with the CYTOP and the PTFE surfaces accounted for the different positive and negative peak voltages. The ripples, shown in Fig. 3b, were due to the damped vibrations of the water drop when it collided with the acrylic walls at each end. Cases involving water drops of different sizes were also studied. Because the contact area was found to be proportional to the volume of the water drop (Supplementary Fig. S2), a linear relationship between the amount of induced charge and the contact area was observed as expected (Fig. 4a). Using Eq. (2), one can estimate the energy harvested per cycle as shown in Fig. 4b. The linear relationship indicates an energy density of 0.36 *μ*J/cm^2^ per cycle.

#### Enhanced performance with a variable capacitor-based scheme

**Figure 5:**
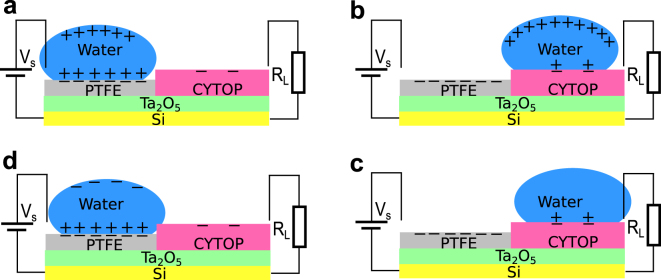
Harvesting energy using a variable capacitor-based scheme. (**a**) External bias applied to the water drop. (**b**) The drop moving to the thicker CYTOP coating. (**c**) Electricity delivered to the load. (**d**) Drop moving back to PTFE.

**Figure 6:**
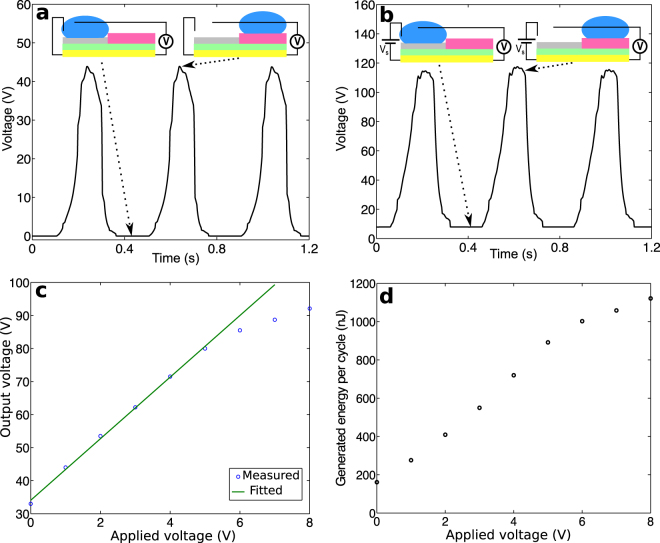
Results from devices using an external bias to enhance performance. (**a**) The open-circuit voltages with a zero external bias, i.e. *V*_*s*_ = 0. (**b**) The open-circuit voltages under an *V*_*s*_ = 8*V* bias. (**c**) Peak voltages versus external bias. (**d**) Energy generated per cycle versus external bias.

The performance of the harvesters can be enhanced if the scheme based on variable capacitors is used with an external bias. In this case, the hydrophobic coatings can be fabricated with significantly different thicknesses. The resulting device essentially behaves as a variable capacitor with the water drop as the moving electrode. In this study, the PTFE and the CYTOP coatings were 0.5 *μ*m and 8 *μ*m, respectively, corresponding to measured capacitances of 3.99 nF and 0.26 nF, respectively. Figure 5 illustrates the working mechanism of this scenario. An external, positive voltage bias is applied when the water drop is on PTFE (Fig. 5a). When the drop moves to the CYTOP side, the electric potential of the drop relative to the substrate increases due to the reduced capacitance and the additional, induced charge (Fig. 5b). The electric potential energy increased accordingly, which is delivered to the resistor upon closing of the circuit (Fig. 5c). When the drop moves back to the PTFE surface, the opposite charge is induced (Fig. 5d) and will be neutralized when the bias is applied again (Fig. 5a). Thus, The measured voltages will always be positive. The open-circuit voltages obtained with this scheme under a 2.5 Hz vibration were measured continuously. They are presented in Fig. 6a for the case of a zero bias and in Fig. 6b for the case of an 8 V bias. In this scenario, because the external bias is applied when the drop is on PTFE, the measured voltage on the PTFE side is the same as the applied bias. Therefore, the voltages shown in Fig. 6a,b are non-negative. For a 400 *μ*L water drop, the peak voltage reached 44 V and 115 V for the two cases, respectively. Note that with a more than 10-fold decrease in the thickness of the CYTOP layer, i.e. from 6.6 *μ*m to 0.5 *μ*m, the increase in voltage was less than 5%, i.e. from 42 V shown in Fig. 3 to 44 V. Because such thickness variation does not change the thickness of the double layer at the interface, the resulting slight change in the peak voltage indicates the electrostatic induction in the silicon substrate was negligible in this case.

**Figure 7:**
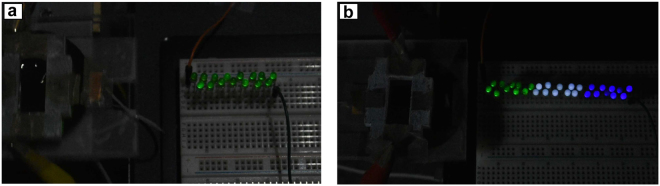
Devices using a 400 *μ*L water drop to illuminate commercial LEDs. (**a**) 15 green LEDs lit a zero external bias. (**b**) 30 LEDs (10 green LEDs, 10 white LEDs, and 10 blue LEDs) lit under an 8 V bias.

The effects of the external bias were studied by using different values in experiments and measuring the open-circuit voltages. The water drop was driven from the PTFE to the CYTOP surface by introducing an 8° inclination. The open-circuit voltages were measured when the drop was on CYTOP. As shown in Fig. 6c, the open-circuit voltage was proportional to the applied bias. It reached 33 V and 92 V for a zero and an 8 V bias, respectively. The peak values presented in Fig. 6a,b are higher because of the overshoot resulted from the collisions of the drop and acrylic wall. Note that not all charge will move with the drop if it moves from one material to the other^21,30,31^. However, if the drop is always discharged before it moves to the other material, the leftover charge from the previous cycle will always be discharged, leaving no effect on the harvest energy as shown in Fig. 3. In this scenario, the drop was discharged only on CYTOP; thus, the leftover charge on PTFE need to be included in the calculation of the energy harvested. Experimental results suggest that the leftover charge is linearly proportional to the applied bias (Supplementary Fig. S3). A parasite capacitor, therefore, can be used to model such a behavior (Supplementary Fig. S4). The net energy harvested per cycle, which is the difference between the total raised potential energy and the initial energy input from the bias, can thus be calculated. The results are shown in Fig. 6d. According to the data collected, a higher bias increases the harvested energy. For example, the energy harvested per cycle increased from 0.16 *μ*J with a zero bias to 1.12 *μ*J with an 8 V bias. Figure 7 demonstrates that the energy harvested from a low-frequency vibration by a water droplet is sufficient to illuminate commercial LEDs. Under a 2.5 Hz vibration, a harvester illuminated 15 commercial LEDs that were connected in series (Fig. 7a, Supplementary Video 1) with a zero bias. Under an 8 V bias, it illuminated 30 commercial LEDs connected in series (Fig. 7b, Supplementary Video 2).

## Discussion

One of the unique features of the proposed approach is that the hydrophobic surface is created with two materials, i.e. PTFE and CYTOP, which acquire very different amount of saturated, negative charge from contact electrification. In particular, a −8.9 nC/cm^2^ difference in surface charge density was obtained, which triggered strong electrostatic induction so that energy could be harvested efficiently as electric potential energy. Furthermore, the electric energy can be delivered to external circuits at its peak value via a simple passive switching mechanism. Because potential energy depends only on the position and not the frequency of the motions of the water drop, this approach is suitable for harvesting energy from low-level, low-frequency excitations. It is worth noting that an 8° inclination is sufficient to drive a 400 *μ*L water drop to move across the two-region surface with a distance of 1.3 cm. The total mechanical potential energy available for harvesting is thus 7.1 *μ*J. As demonstrated in Fig. 4b, a harvesting efficiency of 2.5% has been achieved in this study. The efficiency is independent of the load resistance and is two orders of magnitude higher than the optimal efficiency of 0.011% reported in a previous study for triboelectric generators^19^, in which a 30 *μ*L water droplet harvested 30 nJ from the released potential energy, 265 *μ*J, of the droplet falling from a height of 90 cm. When vibratory excitations are concerned, a 1.5 *μ*W optimal power output has been reported with 1 mL of water (24 droplets, 42 *μ*L each) based on the existing methodology^22^. If driven under the same vibration, our device would produce 27 *μ*W, an 18-fold increase. While nanocomposite materials with improved properties, e.g. MoS_2_/PDMS, can be used to improve the efficiency of existing triboelectric energy harvesting schemes^32^, such improvement is limited by the strength of the electrostatic induction in the substrate.

In summary, electrostatic energy harvesting relies on the work done by the external energy source against electrostatic forces between opposite charges. Therefore, an electrostatic harvester behaves capacitively in nature and prefers more charges to be involved and a wider range of capacitance change. The use of different materials in creating a hydrophobic surface allows for a continuous electrostatic induction process, leading to an alternate current when the water drop is driven across the surface continuously. The performance can be further improved if materials with larger difference in surface charge densities are used. For example, if the hydrophobic surface can be created with PTFE and a material that acquires positive charge through contact electrification, the amount of induced charge in the water drop will greatly increase, resulting in much increased electric potential energy.

As demonstrated, this method can effectively harvest energy from low-level, low-frequency energy sources. It may lead to methods for harvesting energy from motions of liquids ranging from ocean waves^33^ to falling raindrops^34,35^. While a silicon substrate was used as the back electrode in this study, flexible substrates such as graphene can be used instead. With enclosed designs and proper packaging strategies^21,22^, flexible, encapsulated devices can be fabricated. As the method works without an external electrical source, it holds potential in applications such as self-powered wearable electronics^36^.

## Methods

### Device fabrication

Silicon wafers with a 300 nm-thick layer of tantalum pentoxide (Ta_2_O_5_) were used as the substrates for the devices, one device per wafer. Two kinds of devices were fabricated. In the first kind, the thicknesses of the PTFE and CYTOP coatings were fabricated to be similar. The Teflon^©^ AF 2400 solution was first uniformlly applied to one half of the tantalum pentoxide surface. The wafer with the coating was then oven dried at 200 °C for 1 hour. The process was repeated several times so that the desired thickness was obtained. The CYTOP^©^ AF solution was then applied to the other half of the tantalum pentoxide surface in the same manner. The wafer was then cured at 250 °C for one day. The thicknesses were 6.6 *μ*m and 10 *μ*m for the PTFE and CYTOP coatings, respectively. In the second kind of the devices, the PTFE coating was first spin coated onto tantalum pentoxide. The thickness of the coating was 0.5 *μ*m. After the coated wafer was oven dried, a thick layer of CYTOP was applied to half of the PTFE coating to reach a total thickness of 8 *μ*m. The wafer was then cured at 250 °C for one day. Acrylic sheets (12 mm × 25 mm) coated with a commercial super-hydrophobic material (RUST-OLEUM NeverWet) were used as side walls to keep the water drop within the desired area. Tungsten wire leads were used as the metal contacts to avoid chemical reactions with water. Topical subheadings are allowed. Authors must ensure that their Methods section includes adequate experimental and characterization data necessary for others in the field to reproduce their work.

### Measurements

A shaker (Labworks ET-126-1) was used to excite the devices. Measurements were obtained with an electrometer (Keithley 6517B). The parasite capacitance of the Triax cable provided by Keithley Inc. was approximately 300 pF. Cables that avoided such parasite capacitance were fabricated and used to obtain the measurements. The communications between a computer and the electrometer were established with a KUSB-488B cable.

### Calculation of harvested energy per cycle under an external bias

When the water drop moves from Fig. 5c,d, the total charge is *C*_*pp*_ *V*_*s*_ − *Q*_*p*_. After the drop makes the connection with the metal contact on PTFE, the input energy can be expressed as below

3 $${E}_{I}=\frac{1}{2}{C}_{p}{V}_{s}^{2}-sign({C}_{pp}{V}_{s}-{Q}_{p})\frac{{({C}_{pp}{V}_{s}-{Q}_{p})}^{2}}{2{C}_{p}}$$

where *sign*(*x*) is the signum function. When the water drop is on PTFE, the capacitor is charged to *V*_*s*_. While the drop moves to the CYTOP surface, the energy delivered to the resistor can be expressed as,

4 $${E}_{O}=\frac{{({C}_{pr}{V}_{s}+{Q}_{p})}^{2}}{2{C}_{c}}$$

Therefore, the harvested energy per cycle is

5 $$E={E}_{O}-{E}_{I}=\frac{{({C}_{pr}{V}_{s}+{Q}_{p})}^{2}}{2{C}_{c}}+sign({C}_{pp}{V}_{s}-{Q}_{p})\frac{{({C}_{pp}{V}_{s}-{Q}_{p})}^{2}}{2{C}_{p}}-\frac{1}{2}{C}_{p}{V}_{s}^{2}$$

### Data availability

The datasets generated during and analysed during the current study are available from the corresponding author on reasonable request.

## Electronic supplementary material

Supplementary Information

Supplementary Video 1

Supplementary Video 2
