# Supplementary material for: Harvesting energy from low-frequency excitations through alternate contacts between water and two dielectric materials
Source: Sci Rep. 2017 Dec 7;7:17145. doi: 10.1038/s41598-017-17522-8 (PMC5719356; doi:10.1038/s41598-017-17522-8)
Supplement: Supplementary file 1 — Supplementary Information [file 41598_2017_17522_MOESM1_ESM.pdf]

Harvesting energy from low-frequency excitations through alternate contacts between water and two dielectric materials

Jian Yu,[a] Enze Ma,[b] and Tianwei Ma*[c]
[a] Department of Civil & Environmental Engineering,
[b] Department of Neuroscience, Duke University
[c] College of Engineering, University of Hawaii at Mānoa
* E-mail: tianwei@hawaii.edu

Supplementary Materials

Supplementary figures: Fig. S1 to S4
Movies: Video 1 and Video 2

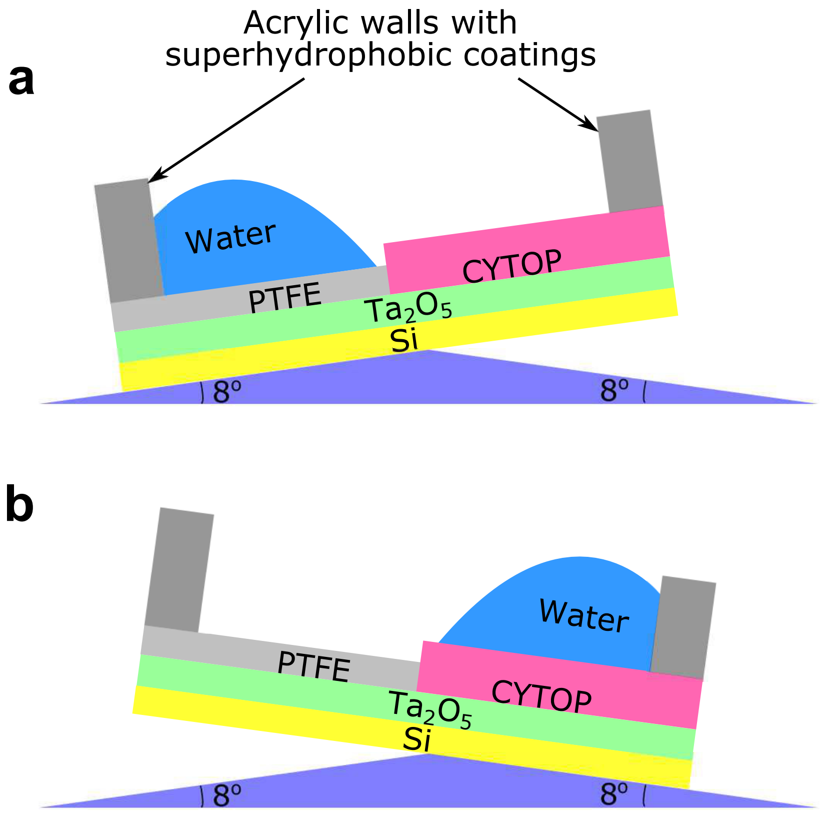

Fig. S1 A prototype generator driven manually on inclined surfaces. **a.** The water drop on PTFE. **b.** The water drop on CYTOP.

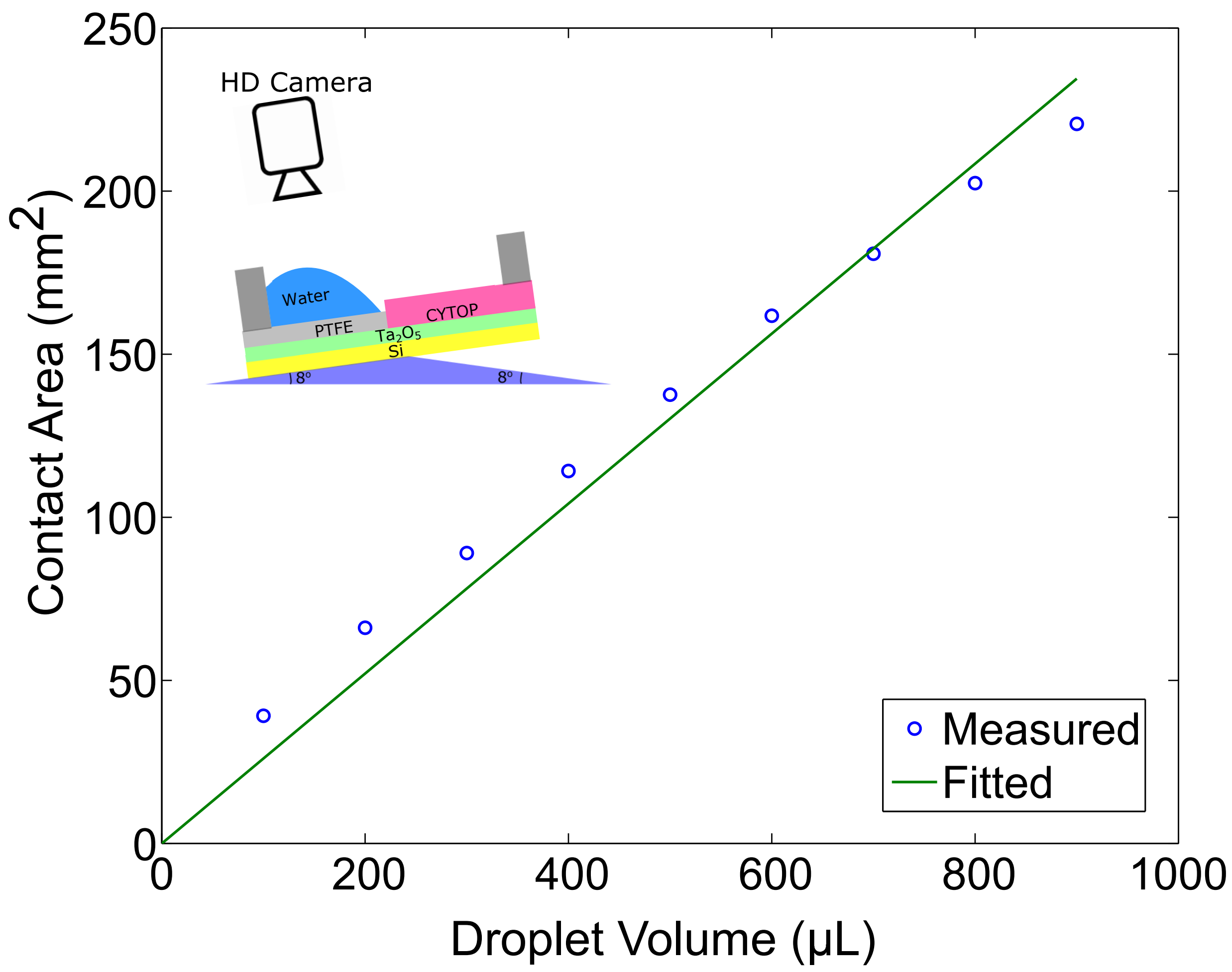

Fig. S2 The relationship between the drop volume and the contact area. The contact area was estimated using the image provided by a HD camera.

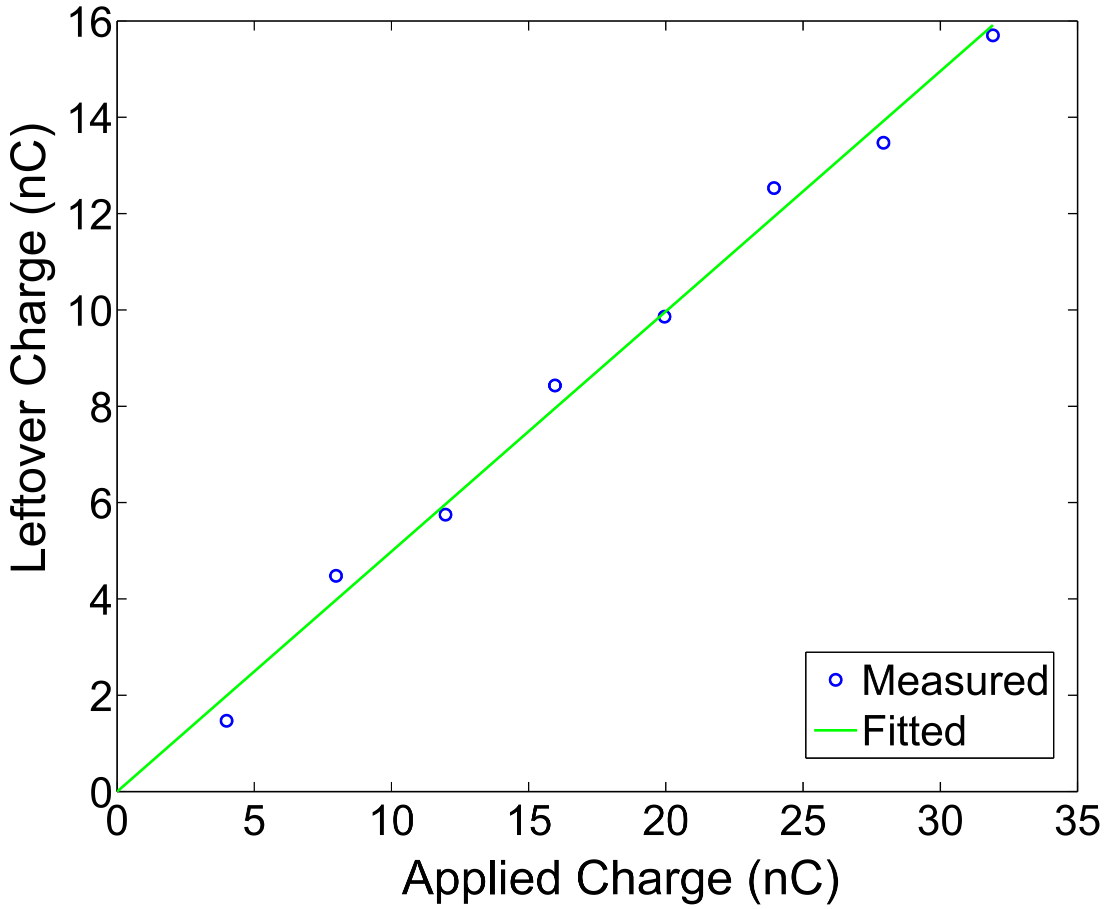

Fig. S3 Charge remained on PTFE after the water drop had moved away. The water drop was first charged by an external voltage source when the drop was on PTFE. The drop was then driven to CYTOP, where it was discharged. Finally, the drop was moved back to PTFE and an electrometer Keithley 6517B was used to measure the charge in the drop. The leftover charge shown in the figure is the sum of the measurement of 6517B and Q_c.

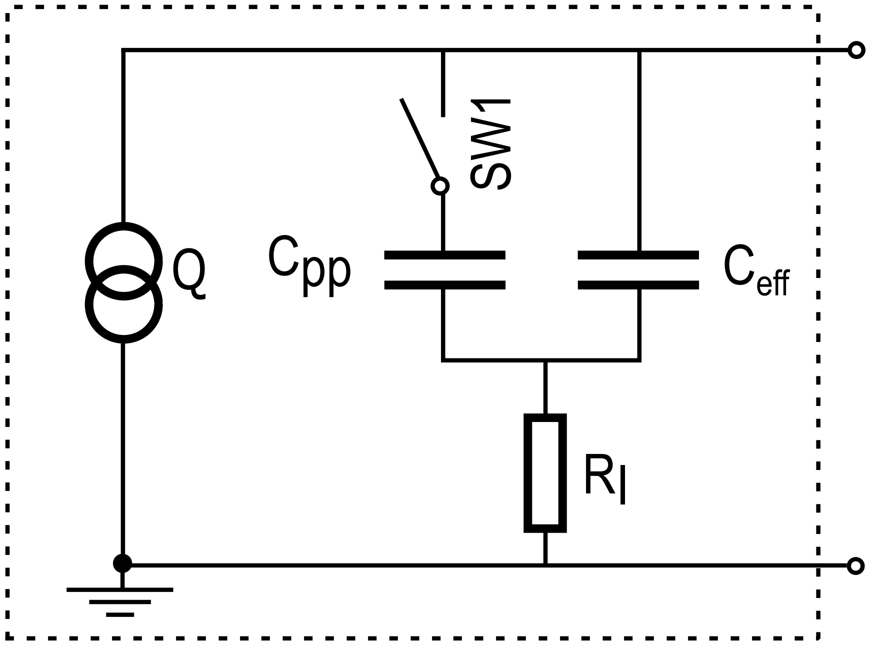

Fig. S4 Circuit model incorporating a parasite capacitor to account for charge left on PTFE. When the water drop is on PTFE, the total capacitance is $C_p = C_{pp} + C_{pr}$, where C_{pp} denotes the parasite capacitance and C_{pr} the actual effective capacitance. When the drop is on PTFE, SW1 is closed to allow C_{pp} to be charged. When the water drop moves away from the PTFE surface, SW1 is open so that the charge in C_{pp} remains on PTFE. The effective capacitance of the water drop is denotd by C_{eff}, which is $C_{eff} = C_{pr}$ when the drop is on PTFE and $C_{eff} = C_c$ when it is on CYTOP.
